# Immunogenicity and protective efficacy of RSV G central conserved domain vaccine with a prefusion nanoparticle

**DOI:** 10.1038/s41541-022-00487-9

**Published:** 2022-06-30

**Authors:** Jennifer N. Rainho-Tomko, Vincent Pavot, Michael Kishko, Kurt Swanson, Darin Edwards, Heesik Yoon, Lilibeth Lanza, Judith Alamares-Sapuay, Robert Osei-Bonsu, Sophia T. Mundle, Dave A. Murison, Scott Gallichan, Simon Delagrave, Chih-Jen Wei, Linong Zhang, Gary J. Nabel

**Affiliations:** 1 Sanofi, 2501 Discovery Drive, Suite 300, Orlando, FL 32826 USA; 2 Sanofi, 1541 Avenue Marcel Mérieux, Marcy l’Etoile, France; 3 Sanofi, 38 Sidney Street, Cambridge, MA 02139 USA; 4 Sanofi, 270 Albany, Cambridge, MA 02139 USA; 5 Sanofi, 95 Willowdale Blvd, Toronto, ON Canada; 6 Present Address: Ring Therapeutics Inc., 620 Memorial Drive, Cambridge, MA 02139 USA; 7 Present Address: Moderna Inc., 200 Technology Square, Cambridge, MA 02139 USA; 8 Present Address: Apellis Pharmaceuticals Inc., 200 5th Ave, Watertown, MA 02451 USA; 9 Present Address: ModeX Therapeutics Inc., 22 Strathmore Rd., Natick, MA 01760 USA

**Keywords:** Adaptive immunity, Protein vaccines

## Abstract

Respiratory syncytial virus (RSV) G glycoprotein has recently reemerged as a vaccine antigen due to its ability to elicit potent neutralizing antibodies and ameliorate disease in animal models. Here we designed three constructs to display the G central conserved domain (Gcc) focused on inducing broad and potent neutralizing antibodies. One construct displaying Gcc from both RSV subgroups trimerized via a C-terminal foldon (Gcc-Foldon) was highly immunogenic in mice and in MIMIC, a pre-immune human in vitro model. To explore an optimal RSV vaccine, we combined the Gcc-Foldon antigen with a stabilized pre-fusion-F nanoparticle (pre-F-NP) as a bivalent vaccine and detected no antigenic interference between the two antigens in the MIMIC model. In RSV-primed macaques, the bivalent vaccine elicited potent humoral responses. Furthermore, both Gcc-Foldon and the bivalent vaccine conferred effective protection against RSV challenge in mice. This two-component vaccine could potentially provide effective protection against RSV infection in humans and warrants further clinical evaluation.

## Introduction

Respiratory syncytial virus (RSV) is a major cause of severe respiratory disease among the high-risk populations of infants and the elderly^1^, contributing to an estimated 14,000 deaths annually in the US among adults over 65 years of age and immunocompromised individuals^2^. Despite the high public health burden, there is currently no licensed vaccine against RSV^3,4^. RSV surface fusion (F) and attachment (G) glycoproteins play important roles in mediating infection and are both major targets of the humoral immune response^5,6^. RSV F is well conserved while G is highly variable, with the two antigenic subgroups of RSV (A and B) being defined primarily by differences in the G protein^7^. RSV F is well studied, and vaccine and therapeutics development efforts have largely focused on RSV F.

These efforts are guided by structure determination studies showing that F exists in at least two conformations: pre- and post-fusion^8,9^. Pre-Fusion (pre-F) is more effective in inducing neutralizing antibodies (NAbs), accounting for >90% of the F-specific neutralizing activity in humans, making it the favored antigen for vaccine development^6^. We previously reported that a structure-based rationally designed F immunogen could optimize antigen presentation and focus antibody (Ab) responses to key epitopes on the pre-F^10^. This protein was fused to ferritin nanoparticles (pre-F-NP) and modified with glycans to mask poorly neutralizing epitopes, further focusing on the Ab response. Immunization with pre-F-NP elicited potent and durable NAb responses in mice and non-human primates (NHPs) as well as in an in vitro human cell system^10^. Despite high levels of NAb induced by pre-F-NP, there remains an opportunity to improve the neutralizing response by eliciting Abs to other viral proteins implicated in viral entry and immunopathogenesis, such as G, which could potentially increase the protective efficacy of the vaccine.

RSV G is a type II membrane protein containing two highly variable mucin-like regions flanking a central conserved domain (Gcc) of ~40 highly conserved amino acids, including 4 invariant cysteines forming a cysteine noose motif with two disulfide bonds^7,11,12^. The RSV G protein has recently reemerged as a potential vaccine target due to several attributes. First, the high conservation of the Gcc has enabled the isolation of human monoclonal Abs (mAbs) that target RSV Gcc and neutralize both RSV A and B replication in human airway epithelial cells (HAE)^5,13,14^, although polyclonal responses induced by natural infection are often subtype specific^15^. The structure of Gcc bound to RSV NAbs defined neutralizing epitopes on the relatively small Gcc domain^12^. Second, Gcc is critical in mediating infection of bronchial airway epithelial cells; initial RSV infection is thought to be mediated primarily by interactions between the CX3C motif of RSV Gcc and CX3CR1 on ciliated airway cells^5,13,14,16^. It has been shown that Gcc-directed Abs not only neutralize RSV on HAE, but also reduce viral loads in animal prophylaxis and treatment models^17,18^. Third, the CX3C motif in the Gcc is potentially involved in altering the host immune response; Gcc-directed Abs appear to ameliorate disease in animal models^18–20^. Therefore, inclusion of a Gcc component in the vaccine could potentially increase immunogenicity and potency^21^ over an RSV F-alone approach.

To investigate the role of Gcc in RSV protection and explore the synergy of combining Gcc with pre-F-NP as a bivalent vaccine for improved efficacy, we designed several Gcc constructs to be cross-reactive with both subgroups and subsequently identified a lead candidate, i.e., Gcc peptides of RSV A and B trimerized via their C-termini by a T4 bacteriophage fibritin (foldon) domain^22^. When the Gcc-Foldon candidate was co-administered with the pre-F-NP as a bivalent vaccine, potent Ab responses against both Gcc and F were detected in murine and NHP models and in the human biomimetic Modular Immune In vitro Construct (MIMIC^®^) system^23^. In addition, the bivalent vaccine protected mice from RSV challenge. These data suggest that Gcc-foldon could be co-administered with pre-F-NP to potentially improve RSV vaccine immunogenicity and protective efficacy in humans.

## Results

### Gcc antigen designs and selection

**Fig. 1:**
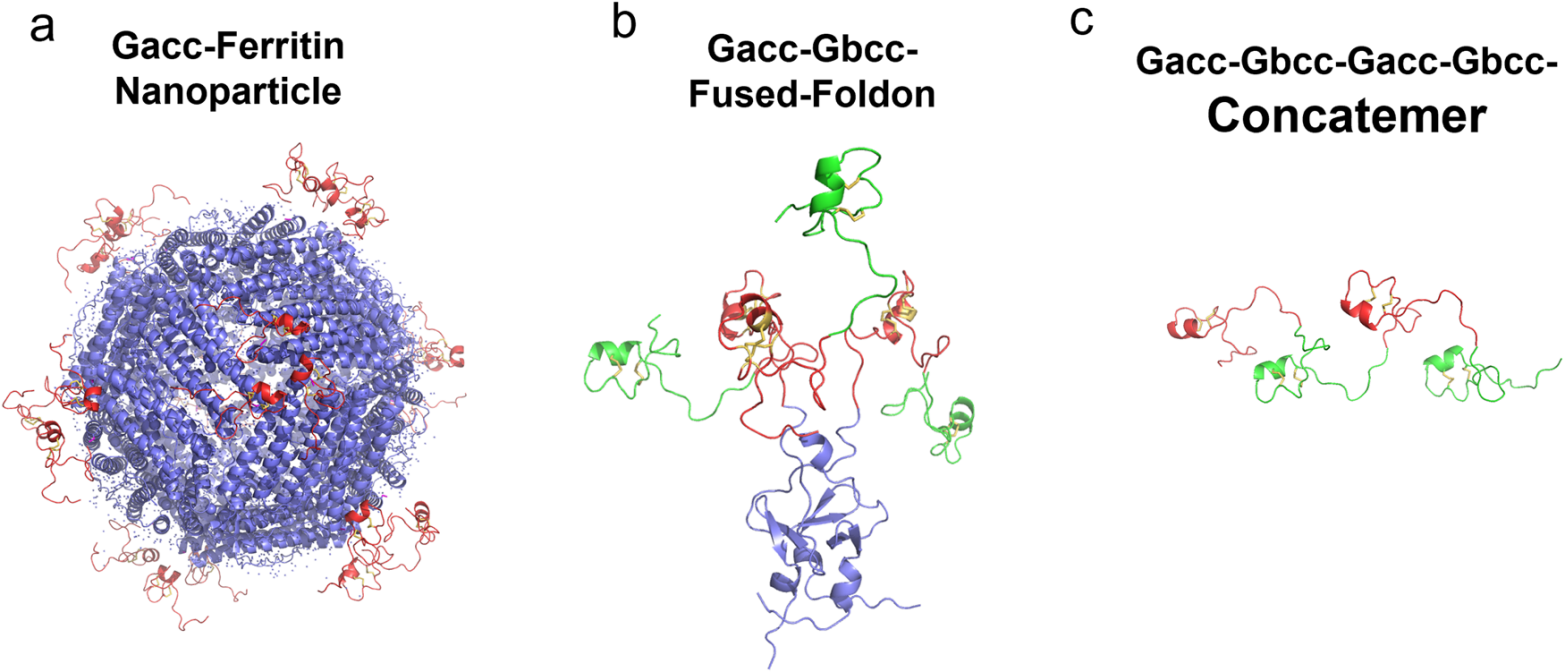
Gcc antigen designs. Peptides corresponding to central conserved regions of RSV G of subgroups A (Gacc, shown in red) and B (Gbcc, shown in green) were **a** bioconjugated to self-assembling ferritin nanoparticles (Gcc-NP), **b** trimerized via a C-terminal foldon domain tandem repeat (Gcc-Foldon), and **c** expressed as concatemers (Gcc-Conc). The foldon and ferritin are shown in blue and the CYS residues in yellow.

Three structure-based designs were utilized to generate antigens corresponding to central conserved regions of RSV G. In the first, peptides corresponding to the Gcc of subgroup A were biochemically conjugated to self-assembling ferritin nanoparticles (Gcc-NP, Fig. 1a) following a method similar to that used to generate RSV pre-F-NP^10^. We had previously demonstrated Gcc from RSV strain A (Gacc) chemically conjugated to ferritin elicited a neutralizing response in mice^15^. In the second, peptides corresponding to Gcc of both RSV subgroups were trimerized via a C-terminal foldon domain tandem repeat (Gcc-Foldon, Fig. 1b). As a third approach, a concatemer consisting of two Gacc and two RSV B Gcc (Gbcc) peptides was constructed (Gcc-Conc, Fig. 1c). The ferritin particle was expressed in *Escherichia coli* and purified using previously described methods^15^. To form Gcc-NP, RSV Gcc peptides were synthesized and biochemically attached to the ferritin particles. Gcc-foldon and Gcc-Conc were expressed in *E. coli*, purified using chelating affinity or AEX columns, respectively, followed by size-exclusion chromatography. The presence of the Gcc antigen was confirmed by binding to the mAb 131–2G^13,24^.

**Fig. 2:**
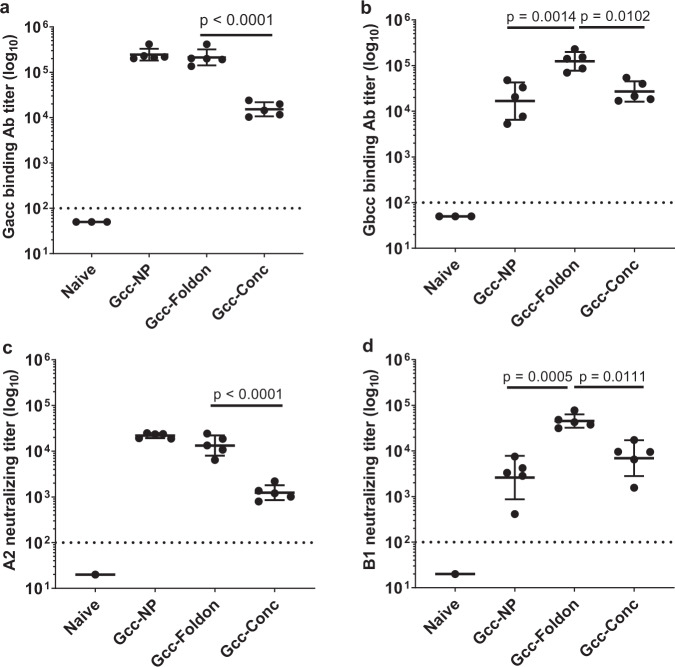
Gcc-foldon induces greatest breadth of antibody responses. Groups of five mice were intramuscularly immunized with 0.5 µg/dose of Gcc-NP and molar equivalents of Gcc peptide of the other antigens formulated with the AF03^33^ squalene-based emulsion adjuvant on days 0, 21, and 42. Blood was collected 14 days after the last immunization. Naive is pooled pre-immune sera. Binding Ab titers were determined by ELISA using plates coated with peptide corresponding to the RSV Gcc of **a** strain A2 or **b** strain B1. NAb titers were determined on HAE cells against RSV strains **c** A2 and **d** B1. Dotted lines represent limits of detection. Error bars represent 1 geometric standard deviation from the geometric mean. All statistical values were performed using Student’s *t*-test.

To identify the optimal Gcc construct, the three designs were first evaluated for breadth and potency of the Ab response by immunizing mice intramuscularly with 0.5 µg/dose on days 0, 21, and 42. Two weeks after the third immunization, G-binding Ab titers were assessed by enzyme-linked immunosorbent assay (ELISA) using plates coated with a peptide corresponding to Gacc or Gbcc. Sera from Gcc-NP and Gcc-Foldon immunized animals exhibited similar binding Ab titers against the Gacc peptide that were ~15-fold higher than those of animals immunized with the Gcc-Conc (*p* < 0.0001, Student’s *t*-test; Fig. 2a). Moreover, Gcc-Foldon immunization induced Gbcc binding Ab titers 7.4- and 4.6-fold higher than Gcc-NP and Gcc-Conc, respectively (*p* < 0.05, Student’s *t*-test; Fig. 2b).

To measure the functionality of the induced anti-Gcc antibodies, an HAE-based neutralization assay previously shown to detect G-specific activity in both Gcc immunized mice and naturally infected humans^15^ was performed. RSV neutralizing titers in sera of the Gcc immunized mice were determined on the HAE cells and closely paralleled the binding Ab titers. Gcc-NP and Gcc-Foldon immunizations induced similarly potent neutralizing activities against RSV A2 (Fig. 2c) that were more than 10-fold higher than those induced by Gcc-Conc (*p* < 0.0001, Student’s *t*-test). Likewise, Gcc-Foldon immunization induced 17.3- and 6.5-fold higher RSV B1 NAb titers than immunization with Gcc-NP or Gcc-Conc respectively (*p* < 0.05, Student’s *t*-test; Fig. 2d). Altogether, Gcc-Foldon immunization elicited the most potent and broad Ab responses in the naive mouse model.

**Fig. 3:**
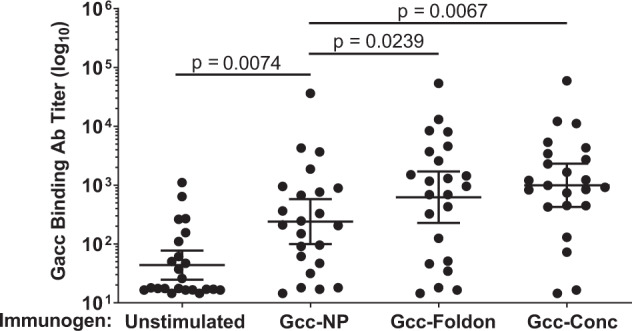
Gcc-foldon and Gcc-Conc elicit higher Gacc-specific Abs than Gcc-NP in MIMIC^®^. Gacc-binding IgG titers were measured after stimulating cells with Gcc-NP, Gcc-Foldon or Gcc-Conc in MIMIC, and determined using the Antibody Forensics technique with Luminex beads coated with peptide corresponding to the RSV Gcc of strain A2. Each dot represents a response from an individual donor, the lines indicate the geometric means, and the error bars represent 95% confidence intervals. *N* = 22. All statistical values were performed using Student’s *t*-test.

To extend the findings in mice, we utilized the MIMIC^®^ human pre-immune in vitro model to evaluate whether pre-existing immunity impacts the immune responses induced by Gcc antigens. By selecting donors with specific immune history, the MIMIC^®^ system can predict how a vaccine candidate will boost immunological responsiveness in a human population^25^. Exposure to the Gcc-NP, Gcc-Foldon, and Gcc-Conc antigens in MIMIC^®^ stimulated higher Gacc-binding Ab titers compared to unstimulated controls (*p* < 0.05, Student’s *t*-test; Fig. 3). Gcc-Foldon stimulated ~2.6-fold higher titers relative to unstimulated controls than molar equivalent doses of Gcc-NP (*p* = 0.0239, Student’s *t*-test; Fig. 3) and a similar difference was observed in Abs targeting G ectodomain (Supplementary Fig. 1). While Gcc-Conc stimulation increased the geometric mean level of Gacc-binding Ab titers slightly more than stimulation by Gcc-Foldon this difference was not significant (Fig. 3). Based on its performance in the mouse and MIMIC^®^ models, Gcc-Foldon was selected as the lead candidate for the remainder of the study.

### Co-administering Gcc-foldon and Pre-F-NP did not result in antigenic interference

**Fig. 4:**
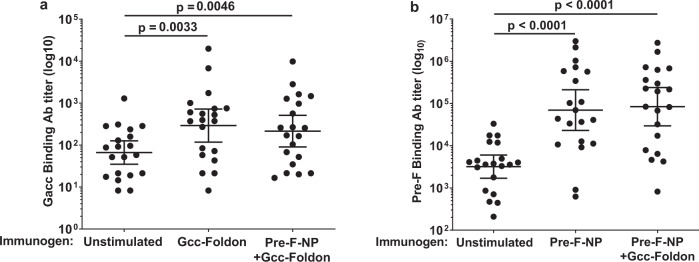
Co-administering Gcc-foldon and Pre-F-NP did not result in antigenic interference in MIMIC^®^. **a** Gcc-specific IgG titers were measured after stimulating cells with Gcc-Foldon or Gcc-Foldon co-administered with pre-F-NP at a 1:1 ratio. **b** Pre-F-specific IgG titers were measured after stimulating cells with pre-F-NP alone or pre-F-NP co-administered with Gcc-Foldon at a 1:1 ratio. Ab binding titers were determined using the Antibody Forensics technique with Luminex beads coated with peptide corresponding to the RSV Gcc of strain A2 or DS-Cav1 (pre-F). In each panel, the immunogen used for stimulation is indicated below the graph. Each dot represents a response from an individual donor, the lines indicate the geometric means, and error bars represent 95% confidence intervals. For comparison, the unstimulated control is labeled as none. *N* = 20. All statistical values were performed using Student’s *t*-test.

Toward designing an optimal vaccine, we evaluated the compatibility of our previously reported pre-F-NP^10^ with the Gcc-Foldon immunogen in the MIMIC^®^ system. Binding Ab titers stimulated by co-administration did not differ significantly from those stimulated by either antigen alone (Fig. 4a, b). Gcc-Foldon increased the level of Gacc-binding Abs by ~4.4-fold over unstimulated controls (*p* < 0.05, Student’s *t*-test; Fig. 4a). Co-administration of Gcc-Foldon and pre-F-NP stimulated similar titers to Gcc alone (Fig. 4a). Stimulation with pre-F-NP increased pre-F-binding Ab titers by ~21.7-fold and similar titers were observed following co-administration of pre-F-NP with Gcc-Foldon (*p* < 0.0001, Student’s *t*-test; Fig. 4b). In summary, co-administration of pre-F-NP and Gcc-Foldon did not interfere with stimulation of Gacc or pre-F binding Abs.

### Co-immunization of RSV-primed NHPs with Gcc-foldon and pre-F-NP robustly boosted humoral responses

**Fig. 5:**
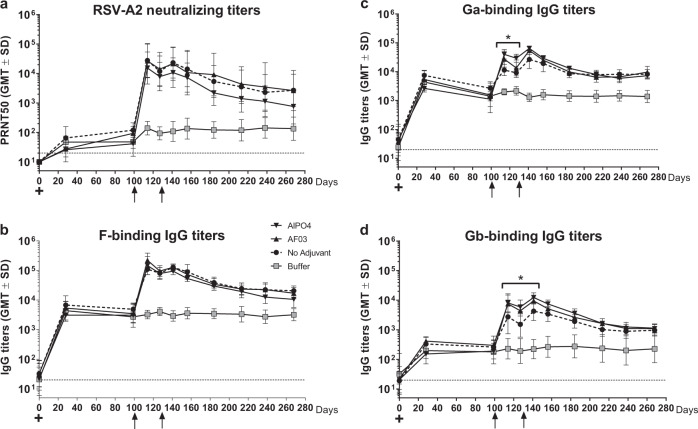
Humoral immune responses to co-administration of pre-F-NP and Gcc-Foldon in RSV-primed cynomolgus macaques. **a** RSV A2 neutralizing titers (PRNT_50_), **b** RSV F-specific IgG ELISA titers, **c** RSV Ga-specific IgG ELISA titers, and **d** RSV Gb-specific IgG ELISA titers following intramuscular vaccination of six cynomolgus macaques/group without adjuvant or with: AlPO4 or AF03 on day 101 and day 129 (*p value <0.01). GMT geometric mean titers, SD geometric standard deviation; dotted lines = limit of quantification; + = RSV infection on day 0; arrows = immunizations. All statistical values were performed using Student’s *t*-test.

Having demonstrated non-interference in the MIMIC^®^ system, we next utilized an RSV-primed NHP model to evaluate the effect of a previous RSV infection on Ab responses induced by the bivalent vaccine. Macaques were infected with live RSV A2 strain by intranasal and intratracheal routes. Immunization of RSV-primed NHPs with adjuvanted or unadjuvanted pre-F-NP and Gcc-Foldon formulations boosted pre-existing RSV-specific NAb, as quantified by 50% plaque reduction neutralization test (PRNT_50_), as well as RSV F-, Ga- and Gb-specific IgG responses in ELISA (Fig. 5). Interestingly, we observed different adjuvant effects on F and G responses in pre-immune monkeys. F-specific NAb and binding IgG titers did not differ significantly between adjuvanted and non-adjuvanted groups; In the AlPO4-adjuvanted group, slightly lower neutralizing titers than in AF03 and non-adjuvanted groups, but this trend was not statistically significant (*p* > 0.05, Student’s *t*-test; Fig. 5a, b). However, both adjuvants induced significantly (*p* < 0.05, Student’s *t*-test) higher G-binding Ab titers than in the non-adjuvanted group at early timepoints post immunization (i.e., day 14 to day 41) although the differences were no longer significant at later timepoints (Fig. 5c, d). In all three groups, the neutralizing and binding Ab titers significantly increased after dose 1 (*p* < 0.001, Student’s *t*-test), peaked 14 days after the second immunization (GMT PRNT_50_: ~10,000; F: ~100,000; Ga: ~10,000 and Gb: 5000) and then declined slowly to reach a plateau approximately 3 months post immunization.

### Gcc-foldon immunization conferred protection against RSV challenge in mice

To investigate the protective efficacy of Gcc-Foldon, mice were immunized on days 0 and 21 with either 1 µg of this antigen or 1 µg of pre-F-NP to serve as the protection benchmark while a third group was immunized with PBS. In addition, the fourth group of mice that received 1 µg each Gcc-Foldon and pre-F-NP was included to determine if the combination of both antigens could increase protection over that provided by the single antigen alone. Each immunogen was formulated with AF03. On day 35, the immunized mice were challenged intranasally with 10^6^ plaque-forming units (PFU) of RSV A2, and their lungs were harvested on day 39 for virus titration.

**Fig. 6:**
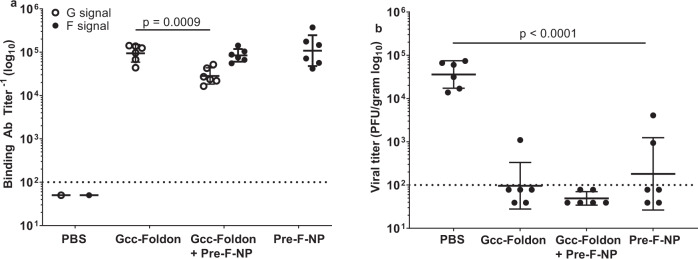
Protective efficacy in mice. Groups of mice were immunized intramuscularly on days 0 and 21 with either 1 µg of pre-F-NP, 1 µg of Gcc-Foldon, with PBS, or 1 µg each of pre-F-NP and Gcc-Foldon. Each immunogen was formulated with AF03. On day 35, mice were challenged intranasally with 10^6^ PFU of RSV A2. Blood was collected on day 35 and the animals sacrificed on day 39. **a** Binding Ab titers were determined in ELISA using plates coated with recombinant RSV F or a 1:1 mixture of RSV Gacc and Gbcc peptides. **b** Viral load in mouse lungs 4 days post challenge was determined by titration on Vero cells. Dotted lines represent limits of detection. Error bars represent 1 geometric standard deviation from the geometric mean. Binding Ab *p* values were derived by Student’s *t*-test. The lung titer *p* values were derived by multiple comparison using Tukey–Cramer HSD.

Bivalent vaccination elicited both F and Gcc-binding Abs, although Gcc-binding Ab titers were 3.3-fold lower than when Gcc-Foldon was administered alone (Fig. 6a). Upon challenge, Gcc-Foldon immunized mice and those that received pre-F-NP exhibited similar levels of partial protection; ~2 orders of magnitude decrease in geometric mean lung viral titers was observed in the pre-F-NP immunized group as compared to controls. Co-administration of both antigens further decreased the viral lung burden 3.7-fold as compared to pre-F-NP immunization alone although this difference did not approach significance (Fig. 6b).

## Discussion

RSV is a major cause of severe respiratory disease in infants and the elderly. Despite decades of intensive effort, an effective vaccine to protect these vulnerable populations remains a major unmet medical need. Historic and ongoing trials have mainly focused on F candidates because of their key role in the generation of NAbs and their high conservation between strains. However, there are still questions as to whether pre-F alone can induce optimal protection and whether anti-G responses can further broaden and/or increase protection efficacy.

Abs targeting the G protein could block the attachment of RSV, potentially acting synergistically with F-elicited Abs that block fusion and reduce the pathogenic and immune-modulatory effects induced by soluble G^26–28^. The G protein as a whole is highly variable, heavily glycosylated, and may elicit antibodies to non-functional epitopes. A few attributes of Gcc, such as conservation between both RSV subtypes, lack of glycans, initiation of RSV infection in vivo, and its CX3C motif’s role in pathogenesis, make it an attractive vaccine antigen. Previous approaches to constructing a G vaccine included presenting G from a single strain or including both subgroup A and B components, with both approaches showing promising efficacy in mice^26,29,30^. It is therefore anticipated that the inclusion of a Gcc component in a pre-F vaccine formulation may increase the breadth of Ab response and protective efficacy through additional mechanisms of neutralization and disease modulation.

To further evaluate cross-reactivity and improved immunogenicity of G, we designed three constructs displaying either Gacc alone (Gcc-NP) or together with Gbcc (Gcc-Foldon and Gcc-Conc). Our initial screen in mice showed that the Gcc-Foldon was the most promising candidate as it elicited the greatest breadth and potency of immunogenicity against both RSV subtypes. Interestingly, the Gacc alone construct, i.e., Gcc-NP induced similarly high RSV A Ab titers as the Gcc-Foldon but lower RSV B titers, suggesting that due to the lack of a Gbcc component it was less effective at inducing Abs against subtype B. The Gcc-Conc induced similar binding Ab titers against Gcc from both RSV subtypes, but the levels were significantly lower than those induced by the Gcc-Foldon. This could be due to inefficient exposure of Gcc epitopes to the immune system by a simple concatemer. Our findings suggest that the presentation of the Gcc peptides as a trimer provides uniformity and/or optimal presentation to the immune system. The foldon trimerization domain we utilized is widely used for pre-F vaccine design with no safety issues^10,31^.

We also evaluated the immunogenicity of the G constructs in MIMIC^®^ to simulate the impact of pre-existing human immunity on eliciting further immunological responses. All three antigens were immunogenic but Gcc-Foldon and Gcc-Conc induced the highest Ab titers, suggesting a greater propensity for boosting pre-existing immunity. Since the Gcc-Foldon showed the best immunogenicity in mice and was capable of boosting Ab responses in pre-immune donors, this construct was selected as our Gcc component lead.

Previously, we reported that a pre-F-NP vaccine-elicited NAbs targeting RSV F in its pre-fusion conformation and induced Ab recall responses in the MIMIC^®^ system^10^. As there is currently insufficient scientific evidence that a pre-F only targeted approach can confer full and lasting protection against RSV^5,21^, the addition of a Gcc component to pre-F-NP vaccine approaches can potentially enhance protection through elicitation of two different neutralization mechanisms, i.e., blocking viral fusion by pre-F- and attachment by Gcc-specific Abs. In addition, NAbs to G could provide additional benefits by moderating disease. Toward the goal of combining pre-F-NP and Gcc-Foldon as a bivalent vaccine, we evaluated the compatibility of the two vaccine antigens by co-administering pre-F-NP and Gcc-Foldon in the MIMIC^®^. Co-administration did not interfere with boosting pre-F or Gcc-specific Ab responses compared to Gcc-Foldon or pre-F-NP alone in cells from individuals with pre-existing immunity, thereby demonstrating the feasibility of a bivalent vaccine.

We further explored the effects of prior RSV infection on macaque humoral responses to our vaccine candidate. We immunized NHPs, which were pre-infected with RSV, with the bivalent pre-F-NP + Gcc-Foldon with or without adjuvant. Significant increases in F- and G-specific binding Ab titers, as well as neutralizing responses were observed. Adjuvants AlPO4 or AF03 resulted in a significant but transient increase in G-specific Ab responses compared to non-adjuvanted formulation early after vaccination and did not impact F-specific Ab responses.

Lastly, we performed a mouse challenge study to determine the protective efficacy of Gcc-Foldon alone or in combination with pre-F-NP. Gcc-Foldon administration did not interfere with the induction of F-specific Abs and was as effective in decreasing lung viral burden post challenge as pre-F-NP immunization. Co-administration of both antigens appeared more protective than either alone, although this trend did not reach statistical significance, possibly due to an insufficiently stringent challenge. These data suggest there may be synergy between these two antigens and warrant further evaluation of this dual-component vaccine. It is likely that in elderly humans the benefits of adding a G component to a pre-F-based RSV vaccine will be clearer than in a naive mouse challenge model, as mice are only semi-permissive for RSV replication^32^.

The data presented here identified an approach to effectively induce potent, cross-NAbs to RSV Gcc and demonstrated that it can be combined with our previously described pre-F-NP vaccine candidate without impacting the generation or activity of pre-F specific immune responses. This bivalent vaccine could be a useful strategy to improve immunogenicity and protection from RSV infection.

## Methods

### Antigen expression and purification

Peptides corresponding to the Gcc of RSV subgroup A were produced by solid-phase synthesis and an azide group was chemically added to the peptide N-terminus. Self-assembling ferritin nanoparticles, with engineered free cysteines on the surface, were expressed in 293 mammalian cells^15^. To form Gcc-NP, the Gcc peptides were biochemically attached via the azide group to the engineered free cysteines on the ferritin nanoparticles via an alkyne-maleimide linker.

To construct Gcc-Foldon, a dimer of Gcc peptides from A and B strains was fused together genetically and followed C-terminally with a foldon tag^22^. Plasmids containing these sequences were synthesized and expressed in *E. coli*. Upon refolding from *E. coli* expression, the foldon tag trimerizes, and the resulting construct presents six copies of the Gcc region (three of A and three of B strain). The cleavable C-terminal HIS_6_-tag was used for purification via chelating affinity followed by size-exclusion chromatography.

To construct Gcc-Conc, four Gcc peptides (in order of A2, B1, A2, and B1) were genetically fused together with glutamate-rich linkers to produce a soluble tetramer of Gcc peptides. The construct was expressed in *E. coli* and purified using an AEX column followed and size-exclusion chromatography.

The presence of the Gcc antigen in all three constructs was confirmed by the ability to bind the 131–2G mAb via Bio-Layer Interferometry on an Octet Red96 instrument.

The pre-F-NP construct uses ferritin nanoparticle technology to display eight pre-F moieties from RSV subgroup A only, and harbors engineered glycosylation sites to mask non-neutralizing epitopes on the protein surface. Pre-F-NP DNA was synthesized and cloned into a mammalian expression vector. The DNA was transfected into CHO cells by electroporation, followed by methotrexate selection to isolate stably transfected cell pools. CHO cell clones expressing RSV pre-F-NP were isolated from a pool by flow cytometric sorting and deposition into 96-well plates at one cell per well. To generate RSV pre-F-NP material from CHO pools and clones, cells were seeded into CD CHO medium (Thermo Fisher Scientific) supplemented with 30% (v/v) EfficientFeed B (Thermo Fisher Scientific) and 4 mM L-glutamine and incubated in suspension culture for 7 days. RSV F nanoparticles were purified by an anionic exchange purification using HiTrap Q columns (GE Healthcare) with tris buffer (pH 8.5) mobile phase eluting with increasing sodium chloride concentration^10^.

Gacc for bioconjugation:

(Maleimide)- GSSGSSEEEGGSRQNKPPNKPNNDFHFEVFNFVPCSICSNNPTCWAICKRIPNKKEEE

Ferritin engineered CYS:

DHAAEEYEHAKKLIIFLNENNVPVQLTSISAPEHKFEGLTQIFQKAYEHEQHISESINNIVDHAIKCKDHATFNFLQWYVSEQHEEEVLFKDILDKIELIGNENHGLYLADQYVKGIAKSRKS

Gcc-Foldon:

MEESEESGGRKNPPKKPKDDYHFEVFNFVPCSICGNNQLCKSICKTIPNKKEEEEESEESGGRQNKPPSKPNNDFHFEVFNFVPCSICSNNPTCWAICKRIPNKKEEESSGGSGGGGSGGGGSGGGGSGSSAIGGYIPEAPRDGQAYVRKDGEWVLLSTFLGSGLEVLFQGPLEHHHHHH

Gcc-Conc:

MEESEESGGRQNKPPSKPNNDFHFEVFNFVPCSICSNNPTCWAICKRIPNKEEEEESEESRKNPPKKPKDDYHFEVFNFVPCSICGNNQLCKSICKTIPNKKEESEESGGRQNKPPSKPNNDFHFEVFNFVPCSICSNNPTCWAICKRIPNKEEEEESEESRKNPPKKPKDDYHFEVFNFVPCSICGNNQLCKSICKTIPNKKEESEESGG

Pre-F-NP:

MELLILKANAITTILTAVTFCFASGQNITEEFYQSTCSAVSKGYLSALRTGWYTSVITIELSNIKENKCNGTDAKVKLIKQELDKYKNAVTELQLLMGSGNVGLGGAIASGVAVSKVLHLEGEVNKIKSALLSTNKAVVSLSNGVSVLTFKVLDLKNYIDKQLLPILNKQSCSISNPETVIEFQQKNNRLLEITREFSVNAGVTTPVSTYMLTNSELLSLINDMPITNDQKKLMSNNVQIVRQQSYSIMSIIKEEVLAYVVQLPLYGVIDTPCWKLHTSPLCTTNTKNGSNICLTRTDRGWYCDNAGNVSFFPQAETCKVQSNRVFCDTMNSRTLPSEVNLCNVDIFNPKYDCKIMTSKTDVSSSVITSLGAIVSCYGKTKCTASNKNRGIIKTFSNGCDYVSNKGVDTVSVGNTLYYVNKQEGKSLYVKGEPIINFYDPLVFPSDEFDASISQVNELINQSLAFINQSDELLSGSGSESQVQQQFSKDIEKLLNEQVNKEMQSSNLYMSMSSWSYTHSLDGAGLFLFDHAAEEYEHAKKLIIFLNENNVPVQLTSISAPEHCFEGLTQIFQKAYEHEQHISESINNIVDHAIKSKDHATFNFLQWYVAEQHEEEVLFKDILDKIELIGNENHGLYLADQYVKGIAKSRKS.

### Viruses and cells

Vero-81 (African green monkey kidney epithelial cells, ATCC CCL-81) cells were obtained from the American Type Culture Collection and propagated in Dulbecco’s Modified Eagle Medium (DMEM) supplemented with 10% fetal bovine serum (FBS), L-glutamine and antibiotics.

Recombinant RSV strain A2 (subgroup A) expressing modified Katushka 2 (mKate) was licensed by Dr Marty Moore at Emory University. RSV B1 was obtained from the American Type Culture Collection. The viruses were propagated in Vero-81 cells and titrated using standard protocols^15^.

HAE cells were obtained from LifeLine Cell Technology, CA, USA, and grown and differentiated in a humidified atmosphere (5% CO_2_, 37 °C). On reaching 75–80% confluence in T75 tissue culture flasks fed with a small airway growth medium (Lonza cat. CC-3281,) supplemented with growth factors and hormones (Lonza cat. CC-4538), the cells were dissociated with trypsin/EDTA and seeded on the semipermeable membrane of transwell culture inserts (6.5 mm diameter, 0.4 μm pore size; Corning-Costar) coated with rat tail collagen type 1 (BD Biosciences). When confluence was reached, an air-liquid interface (ALI) was created to trigger differentiation by removing the growth medium from the apical compartment of the culture inserts set in 24-well plates and replacing the growth medium in the basal compartment with a differentiation medium (Lonza cat. CC-3281) supplemented with the differentiation inducer (Lonza cat. CC-4538). Thereafter, the differentiation medium was changed in the basal compartment every other day, and the apical compartment was gently washed with the culture medium once or twice a week to remove accumulated debris and mucus. The cells fully differentiate in 21–25 days of ALI culture into ciliated cells, goblet cells, basal cells, and non-ciliated columnar cells.

### Mouse statement of ethics

The studies were carried out in strict accordance with the recommendations in the Guide for the Care and Use of Laboratory Animals of the National Institutes of Health. The protocols were approved by the Sanofi Institutional Animal Care and Use Committees (Protocol Number 18/0346 for Immunogenicity assessment and 17/0308 for RSV challenge).

### Mouse immunogenicity assessment

Female BALB/c mice were intramuscularly immunized with 0.5 µg/dose of Gcc-NP and molar equivalents of the Gcc peptide of the other antigens on weeks 0, 3, and 6. Each group contained five mice. Antigens were prepared by mixing 50 µL of antigen solution with 50 μL of the 2.5% squalene-based adjuvant AF03^33^ just before immunization of 50 μL into each hind leg. Blood was collected 1 day before the first (for control samples) and 2 weeks after the last immunization.

### Mouse RSV challenge

Female BALB/c mice were intramuscularly immunized on days 0 and 21 with either 1 µg of pre-F-NP^10^, 1 µg of Gcc-Foldon or 1 µg each of pre-F-NP and Gcc-Foldon, while one group received PBS (Fig. 6). Each group contained six mice. Each immunogen was formulated 1:1 with AF03. On day 35 the mice were challenged intranasally with 10^6^ PFU of RSV A2. Blood was collected just before the first immunization on day 0 and on day 35. On day 39 the mice were sacrificed, and their lungs harvested.

### Macaque statement of ethics

Twenty-four, 3-year-old, female cynomolgus macaques (*Macaca fascicularis* – Noveprim – Mauritius) 2.7–5 kg, were group-housed at Sanofi (Marcy l’Etoile – France) in large stainless cages with an automatic watering system and were fed with dry monkey chow (Primate Maintenance Diet 107, SAFE, Route de Saint Bris, 89290 Augy, France) and fresh fruits or vegetables daily. They were provided with behavioral enrichment toys and treats daily. Their health status was determined based on a complete physical examination, tuberculin skin tests, serology screening (α-herpes virus, SIV, filovirus, HAV, HBV), fecal examination, and culture for parasites and enterobacteria screening.

The study was reviewed by the Ethics Committee #11 of Sanofi and the project was approved under MESR number APAFIS#5481–2016052710357414 v3. All experiments were conducted following the European Directive 2010/63/UE as published in the French Official Journal on February 7, 2013.

### Macaque RSV inoculations, immunizations, and samplings of cynomolgus macaques

Macaques were randomized into four groups of six animals each. On day 0, all animals were first sedated (ketamine and midazolam IM), then inoculated intranasally (i.n.) and intratracheally (i.t.) with a dose of human RSV A2 strain (ATCC) of 10^6^ PFU suspended in PBS. For each animal, 100 µL of inoculum was deposited in each nostril with a micropipette and appropriate plastic tips, and 1 mL of inoculum was sprayed in the trachea using an aerosolizer mounted on a safety syringe (Penn-century, MicroSprayer^®^ Aerosolizer, Model IA-1B). Following inoculation, all animals were sat upright with their head tilted slightly backward for at least 10 min.

After RSV infection, macaques were left resting for 3 months before performing the immunizations. Adjuvanted or non-adjuvanted Pre-F-NP and Gcc-foldon formulations (50 µg each) were administered intramuscularly (500 µL) into the deltoid muscle 28 days apart. Two adjuvants were tested: a squalene-based oil-in-water emulsion AF03 (2.5% squalene) and AlPO4 (330 µg Al^+^). Antigen and adjuvant stock solutions were sterilized by sterile filtration on a 0.22 µm pore membrane and characterized for low endotoxin content (<5 IU/mL) by using Endosafe^®^ cartridges and an Endosafe®-PTSTM spectrophotometer (Charles River Laboratories International, Inc., Wilmington, MA).

Adjuvanted formulations were prepared by mixing extemporaneously, under a laminar flow hood, one volume of a two-fold concentrated adjuvant solution with one volume of a two-fold concentrated antigen solution. Before immunization, pH was measured using a Mettler Toledo pH meter and osmolality was measured using a Fiske^®^ Model 210 Micro-Osmometer Fiske Associates apparatus.

Sera were collected at baseline and at different timepoints over 6 months post-dose 1 to assess humoral responses, including RSV A2 NAbs by a plaque reduction neutralizing test (PRNT_50_), F-specific, and G-specific IgG by ELISA.

### MIMIC^®^ analysis

Apheresis blood products were collected from healthy human donors. The collections and study protocol were reviewed and approved by Chesapeake Research Review Inc (Columbia, Maryland) under IRB 0906009. Donor plasma and peripheral blood mononuclear cells were cryopreserved and stored for an extended period of time in vapor phase nitrogen tanks.

To evaluate humoral responses to RSV F and G candidates, differentiated donor antigen-presenting cells (APCs) were stimulated with either untreated (no antigen-stimulated control wells) or pre-pulsed with Gcc-NP, Gcc-Foldon, Gcc-Conc, pre-F-NP or Gcc-Foldon with pre-F-NP at a 1:1 ratio. After priming with antigens, APCs were co-cultured with enriched autologous CD4^+^ T cells and pre-treated CD19^+^ B cells for 13 days and culture supernatants were collected for detection of anti-Pre-F, Gacc, and Gbcc IgG antibodies.

### Mouse RSV F-, Gacc-, Gbcc-, and Gcc-specific IgG ELISAs

Specific IgG was tested by ELISA. Briefly, microtiter plates were coated with 1 µg/mL of recombinant RSV F (SinoBiologicals, cat. #11049-V08B), or a 1:1 mixture (0.5 µg/mL each) of peptides corresponding RSV Gcc from subgroup A2 (amino acid sequence RQNKPPNKPNNDFHFEVFNFVPCSICSNNPTCWAICKRIP) and B1 (amino acid sequence RKNPPKKPKDDYHFEVFNFVPCSICGNNQLCKSICKTIP) in PBS, or 1 µg/mL of each peptide. Plates were incubated overnight at 4 °C and then blocked with PBS-Tween 0.05%-BSA 1% for 1 h.

Sera were three-fold serially diluted in PBS-Tween 0.05%-BSA 1% in the coated plate. After 1 h incubation at room temperature (RT) plates were washed (PBS-Tween 0.1%) and incubated for 1 h at RT with a donkey anti-mouse IgG-HRP (Jackson Immunoresearch, cat. 715–035–151) diluted at 1:10,000. After washing, plates were incubated with 3,3,5,5-tetramethylbenzidine (TMB) substrate (Fisher Scientific, cat. 34028) for 6 min in the dark at RT. Colorimetric reaction was stopped with Thermo Scientific™ Pierce™ Stop Solution for TMB (Fisher Scientific, cat. N600). Optical densities (OD) were measured at 450 nm on an Envision plate reader (PerkinElmer). IgG titers were quantified through interpolating the serum dilutions at OD 0.2 via 4-parameter logistic regression.

### NHP systemic RSV F-, Ga, and Gb-specific IgG ELISAs

Specific IgG responses in NHPs were tested by ELISA. Briefly, microtiter plates were coated with 1 µg/mL of recombinant RSV F, Ga (RSV A, rsb1734) or Gb (RSV B1) glycoproteins (SinoBiologicals, cat. #11049-V08B, #11070-V08H and #13029-V08H respectively) in bicarbonate buffer (Sigma, cat. C3041). Plates were incubated overnight at 4 °C and then blocked with PBS-Tween 0.05%-milk 5% for 1 h.

Sera were two-fold serially diluted in PBS-Tween 0.05%-milk 5% in the coated plate. After 1.5 h incubation at 37 °C, plates were washed (PBS-Tween 0.05%) and incubated for 1.5 h at 37 °C with a goat anti-monkey IgG-HRP (Bio-Rad, cat. AAI42P) diluted at 1:10,000. After washing, plates were incubated with TMB substrate (Tebu-bio, cat. TMB100–1000) for 30 min in the dark at RT. Colorimetric reaction was stopped with 1 N HCl (VWR Prolabo, cat 30024290). OD were measured at 450–650 nm on a Versamax plate reader (Molecular Devices). IgG titers were quantified through an internal human RSV^+^ serum reference. The titer of this reference was previously calculated as the reciprocal dilution to obtain an OD of 1.

### Antibody forensics

Culture supernatants from MIMIC^®^ studies were collected on day 13 for detection of anti-pre-F, anti-Gacc, and anti-Gbcc IgG by a multiplexed Luminex-based method known as Antibody Forensics. Fluorescent carboxylated magnetic beads (Luminex) were activated and coated directly with 2 µg/mL DS-Cav-1 protein, or the peptide corresponding to the RSV Gcc from subgroup A2 or B1 in PBS. The antigen-loaded Luminex beads were thereafter incubated with MIMIC^®^ supernatants and analyzed on the Bioplex-100 system (Bio-Rad).

### Neutralizing antibody titer analyses on Vero cells

RSV A2 50% plaque reduction titers (PRNT_50_) were determined. Briefly, Vero cells were seeded at 25,000 cells/well in 100 μL in 96-well plates suitable for fluorescence reading (Thermo Scientific, cat. 165305) 1 day prior to infection. On the day of infection, serum samples were heat-inactivated and initially diluted 1:10, and two-fold serially diluted in virus growth medium (VGM) containing DMEM (Gibco, cat. 31980–022), 1% Penicillin – Streptomycin (Gibco, cat. 15140–122), and 2% of fetal calf serum (Hyclone, cat. SH30084–04).

RSV A2 (strain ATCC-VR-1540) was diluted to 6000 PFU/mL in VGM without complement and added at 1:1 ratio to diluted sera and incubated 1 h at 37 °C. The Vero cell monolayers were then infected with 50 μL/well of serum/virus mixture (targeting 150 PFUs/well). After centrifugation of the plates for 10 min at 700 × g, the infected cells were incubated 1 h at 37 °C, 5% CO_2_. Then, the supernatant was removed and replaced by a methylcellulose overlay. Plates were subsequently incubated at 37 °C, 5% CO_2_ for 40 h. Following this incubation, plates were fixed with 85% acetone for 1 h at 4 °C. Plates were washed and blocked with PBS plus 1% non-fat dry milk for 1.5 h at 37 °C and then immunostaining was performed with an anti-RSV fusion protein mAb (Abcam, cat. 24011) diluted at 1:1000 for 1.5 h at 37 °C. After washes, a goat anti-mouse IgG PE (Invitrogen, cat. P852) diluted at 1:200 was added for 1.5 h at 37 °C. Plaques were detected and counted in a multi-modal reader (EnSight, PerkinElmer). The 50% neutralizing titers were calculated using a 4-parameter titer logistic regression.

### Neutralizing antibody titer analyses on HAE cells

To determine NAb titers against RSV strains A2 and B1 on HAE cells, serial dilution series of heat-inactivated serum was combined 1:1 with RSV stocks and incubated for 1.5 h at 37 °C. The virus-serum mixtures were added to fully differentiated HAE cells and incubated for 1.5 h at 37 °C. Following incubation, cells were washed to remove the unbound virus and incubated a further 20 h at 37 °C. RSV A2 infection events were counted on a fluorescent microscope. To detect infection with RSV B1, following the 20-h incubation step cells were washed to remove mucus, fixed, permeabilized, and blocked. Cells were then stained with a mouse anti-RSV mAb mixture at 37 °C for 4 h, washed, and stained with Goat anti-mouse IgG conjugated to Alexa Fluor Plus 488 overnight at 4 °C. The next morning, the cells were washed, and infection events were counted on a fluorescence microscope. Results were reported as PRNT_50_.

### Viral titration of mouse lung homogenates

Mouse lungs were harvested, weighed, and placed into M Tubes (Miltenyi Biotec, cat. 130093236) containing 6 mL of L15 medium (Invitrogen, cat. 11415) supplemented with 1% Antibiotic-Antimycotic (Gibco, cat. 15240096) and 1x SPG buffer and homogenized in a gentleMACS™ Octo Dissociator (Miltenyi Biotec). The homogenates were centrifuged at 300 g, 4 °C, for 5 min and the clear phase was collected. The clarified homogenates were 4-fold serially diluted in DMEM supplemented with 1% GlutaMAX (Gibco, cat. 35050079) and 1% Antibiotic-Antimycotic, and 200 µL of each dilution were added to duplicate wells of 24-well tissue culture plates containing 90% confluent Vero cells. The inoculated plates were incubated for 1 h at 37 °C with gentle agitation every 15 min. Inoculum was then removed by aspiration and the cells overlayed with 1 mL of 0.75% methylcellulose in DMEM supplemented with 2% FBS, 2% GlutaMax, and 2% Antibiotic-Antimycotic, and returned to the 37 °C incubator for 5 days to allow plaques to form.

To visualize the plaques, the overlay was removed, and the Vero monolayers fixed with Methanol for 1 h at RT, then washed with water and blocked with PBS + 5% non-fat dry milk for 30 min at RT with gentle agitation. Blocking solution was then replaced with PBS + 1% non-fat dry milk containing a 1:2000 dilution of Anti-RSV F Ab (Abcam, cat ab24011), and the plates immunostained for 1 h at RT with gentle agitation. The plates were then washed with water and further stained with PBS + 1% non-fat dry milk containing a 1:2000 dilution of Goat Anti-Mouse IgG-HRP (Abcam, cat. ab6789) for 1 h at RT with gentle agitation. The plates were again washed with water and the immunostaining developed with TrueBlue Peroxidase Substrate (KPL, cat. 50-78–02). The plaques were counted under a dissecting microscope and the viral titers were reported as PFU per gram of lung tissue.

### Statistics

Statistics were computed with Prism version 6.0 (GraphPad), SAS version 13, and JMP versions Pro 13 and 15. For mouse, NHP, and MIMIC^®^ studies, the *p* values were derived by Student’s *t*-test. The mouse protection study lung titer *p* values were derived by multiple comparison using Tukey–Cramer HSD.

### Reporting summary

Further information on research design is available in the Nature Research Reporting Summary linked to this article.

## Supplementary information

Reporting Summary

Supplementary Figures

## Data Availability

The materials that support the findings of this study are available from the corresponding author upon request. All data needed to evaluate the conclusions in this paper are present in the paper or the Supplementary Materials.
