# Supplementary material for: Immunogenicity and protective efficacy of RSV G central conserved domain vaccine with a prefusion nanoparticle
Source: NPJ Vaccines. 2022 Jun 30;7:74. doi: 10.1038/s41541-022-00487-9 (PMC9244890; doi:10.1038/s41541-022-00487-9)
Supplement: Supplementary file 2 — Supplementary Figures [file 41541_2022_487_MOESM2_ESM.pdf]

Supplemental Figures

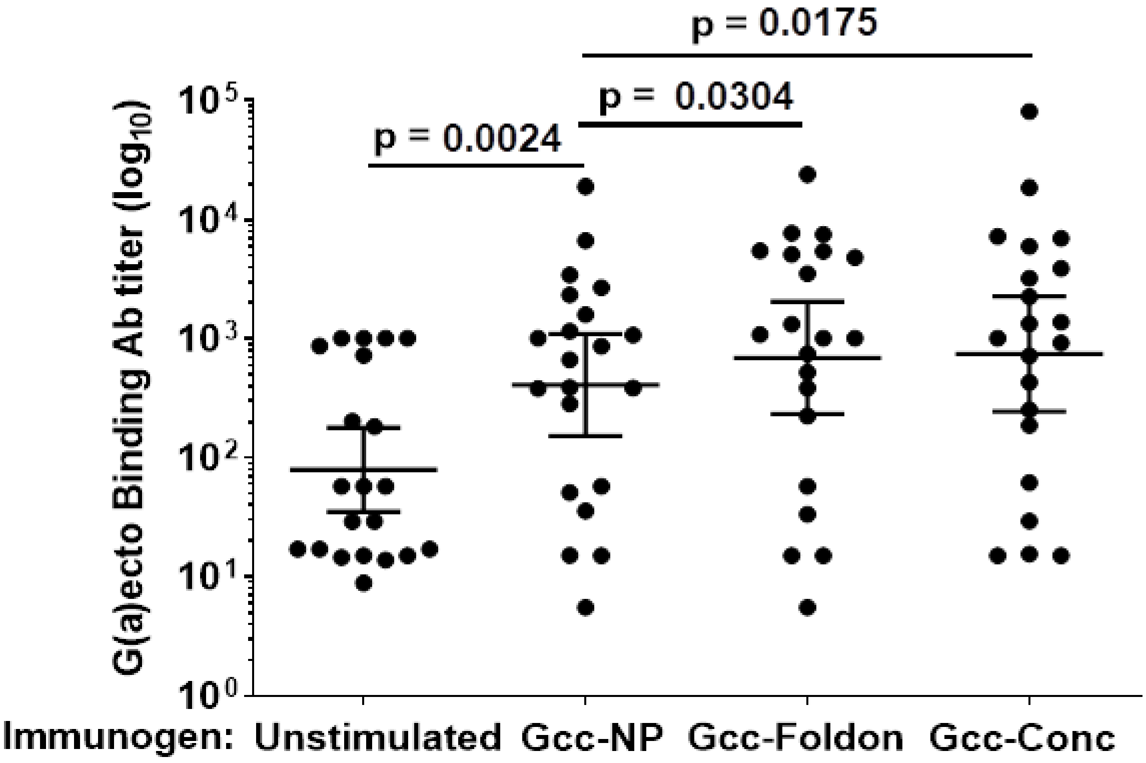

Supp Fig 1. Gcc-NP Foldon elicits lower Abs to the ectodomain of G than Gcc-Foldon or Gcc-Conc in the MIMIC® system. G(a)ecto-binding titers were measured after stimulating cells with Gcc-NP, Gcc-Foldon or Gcc-Conc in MIMIC® and determined using the Antibody Forensics technique with Luminex beads coated with the ectodomain of RSV G of strain A2. Each dot represents a response from an individual donor and the bar indicates the geometric mean and error bars represent 95% confidence intervals. For comparison, the untreated control is labeled as none. N = 21. All statistical values were performed using the Student's *t*-test.
